# Supplementary material for: An integrated three-tier trust management framework in mobile edge computing using fuzzy logic
Source: PeerJ Comput Sci. 2021 Sep 15;7:e700. doi: 10.7717/peerj-cs.700 (PMC8459791; doi:10.7717/peerj-cs.700)
Supplement: Supplemental Information 1 [file peerj-cs-07-700-s001.docx]

[System]

Name='Trust Evaluation1'

Type='mamdani'

Version=2.0

NumInputs=3

NumOutputs=1

NumRules=0

AndMethod='min'

OrMethod='max'

ImpMethod='min'

AggMethod='max'

DefuzzMethod='centroid'

[Input1]

Name='SLA'

Range=[0 100]

NumMFs=3

MF1='low':'trapmf',[-45 -5 20.2380952380952 40.9]

MF2='medium':'trapmf',[28.9354497354497 44.7354497354497 54.7354497354497 70.2380952380952]

MF3='high':'trapmf',[60.1851851851852 85.6 105 145]

[Input2]

Name='Processing_Performance'

Range=[0 100]

NumMFs=3

MF1='low':'trapmf',[-35.7 -3.74 19.973544973545 38.8]

MF2='medium':'trapmf',[29.2 46.3 54.3 70.2380952380952]

MF3='high':'trapmf',[60.7 85.05291005291 104 136]

[Input3]

Name='Violations'

Range=[0 100]

NumMFs=3

MF1='low':'trapmf',[-45 -5 20.2380952380952 40.9]

MF2='medium':'trapmf',[29.2 45 55 70.2380952380952]

MF3='high':'trapmf',[60.4497354497354 95 105 145]

[Output1]

Name='output1'

Range=[0 1]

NumMFs=3

MF1='mf1':'trimf',[-0.4 0 0.4]

MF2='mf2':'trimf',[0.1 0.5 0.9]

MF3='mf3':'trimf',[0.6 1 1.4]
