# Supplementary figures and images for: An integrated three-tier trust management framework in mobile edge computing using fuzzy logic
Source: PeerJ Comput Sci. 2021 Sep 15;7:e700. doi: 10.7717/peerj-cs.700 (PMC8459791; doi:10.7717/peerj-cs.700)

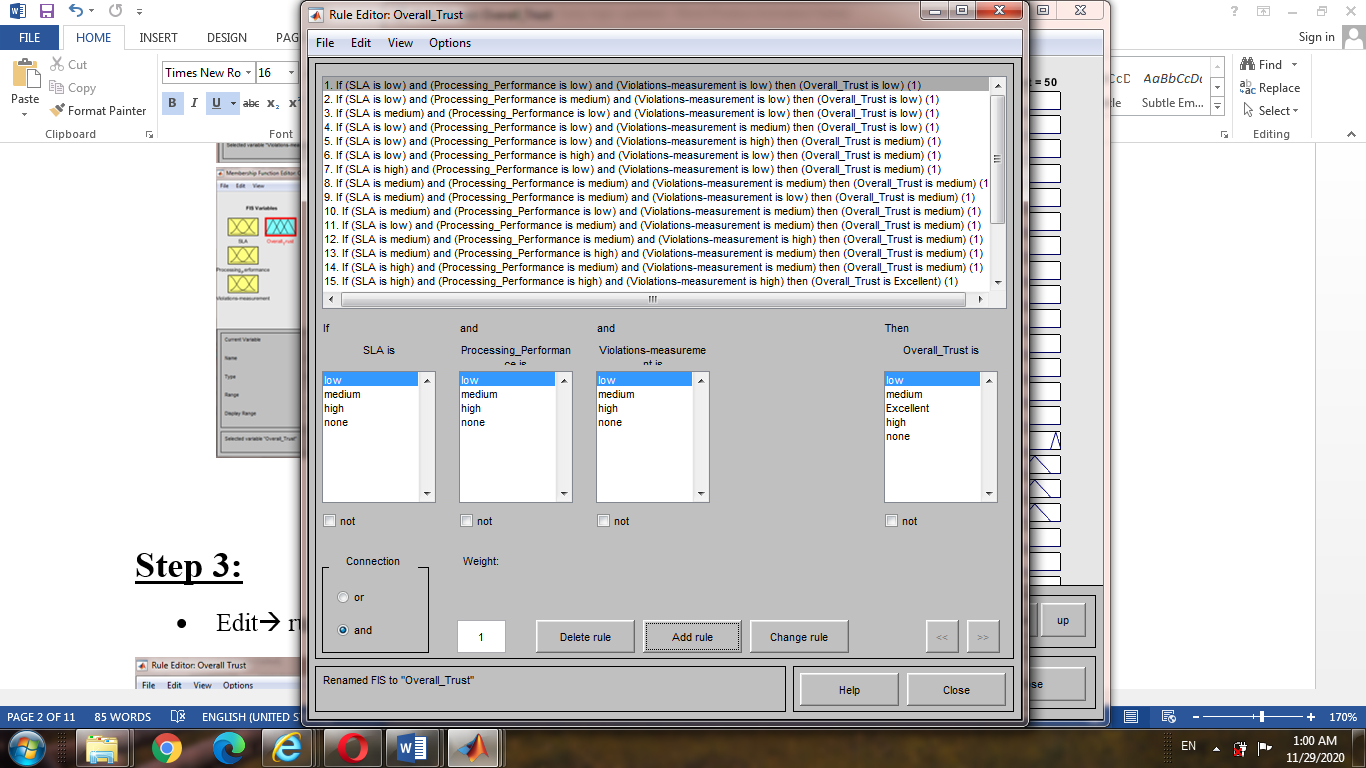


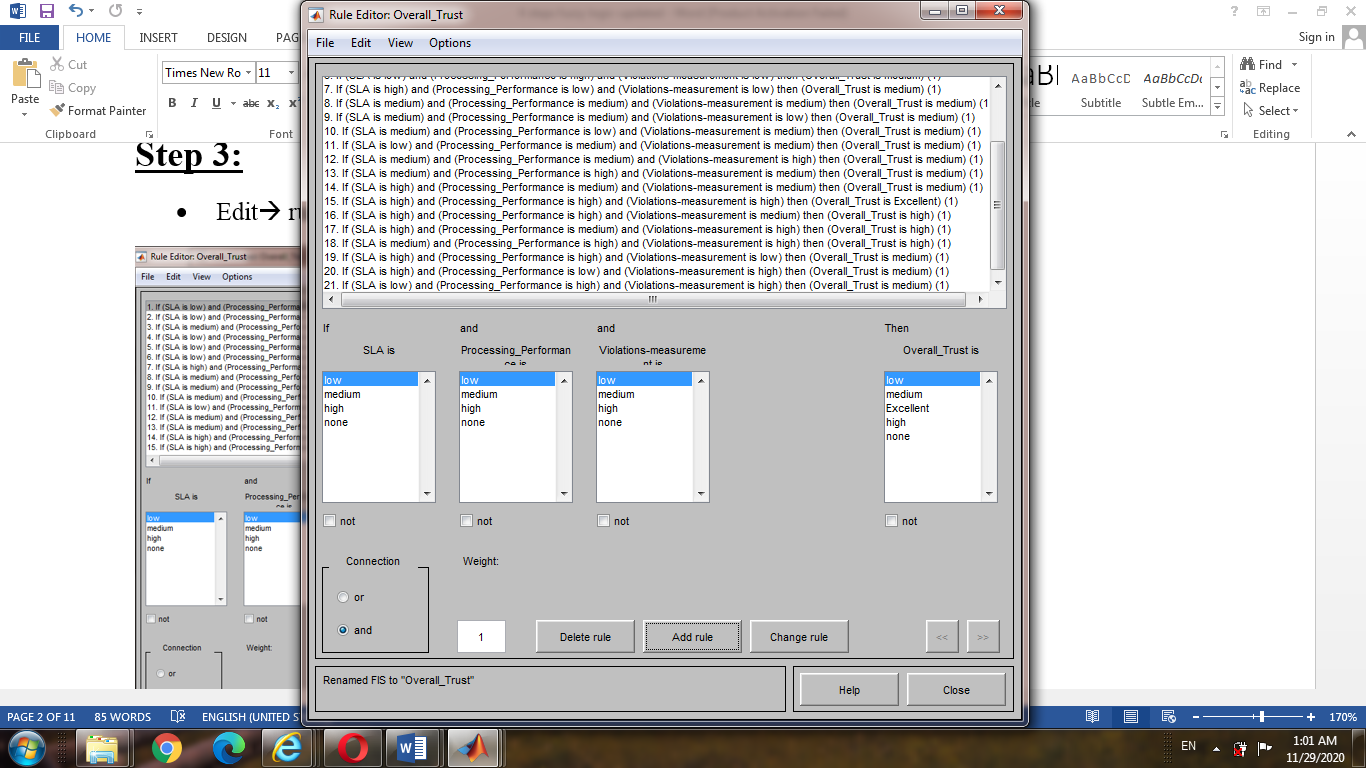

Supplement: Supplemental Information 2 [file peerj-cs-07-700-s002.docx]
